# 3D-XGuide: open-source X-ray navigation guidance system

**DOI:** 10.1007/s11548-020-02274-0

**Published:** 2020-10-15

**Authors:** Ina Vernikouskaya, Dagmar Bertsche, Wolfgang Rottbauer, Volker Rasche

**Affiliations:** Clinic of Internal Medicine II, Ulm University Medical Center, Albert-Einstein-Allee 23, 89081 Ulm, Germany

**Keywords:** Open-source software, Image-guided interventions, X-ray fluoroscopy, Image fusion, Multimodal visualization

## Abstract

**Purpose:**

With the growing availability and variety of imaging modalities, new methods of intraoperative support have become available for all kinds of interventions. The basic principles of image fusion and image guidance have been widely adopted and are commercialized through a number of platforms. Although multimodal systems have been found to be useful for guiding interventional procedures, they all have their limitations. The integration of more advanced guidance techniques into the product functionality is, however, not easy due to the proprietary solutions of the vendors. Therefore, the purpose of this work is to introduce a software system for image fusion, real-time navigation, and working points documentation during transcatheter interventions performed under X-ray (XR) guidance.

**Methods:**

An interactive software system for cross-modal registration and image fusion of XR fluoroscopy with CT or MRI-derived anatomic 3D models is implemented using Qt application framework and VTK visualization pipeline. DICOM data can be imported in retrospective mode. Live XR data input is realized by a video capture card application interface.

**Results:**

The actual software release offers a graphical user interface with basic functionality including data import and handling, calculation of projection geometry and transformations between related coordinate systems, rigid 3D-3D registration, and template matching-based tracking and motion compensation algorithms in 2D and 3D. The link to the actual software release on GitHub including source code and executable is provided to support independent research and development in the field of intervention guidance.

**Conclusion:**

The introduced system provides a common foundation for the rapid prototyping of new approaches in the field of XR fluoroscopic guidance. As a pure software solution, the developed system is potentially vendor-independent and can be easily extended to be used with the XR systems of different manufacturers.

## Introduction

With the increased complexity of transcatheter interventions, the demand for improved guidance and navigation is steadily rising. X-ray (XR) fluoroscopy is the conventional modality that is used for guiding these procedures, primarily due to its real-time imaging capability and excellent visualization of the medical devices (catheters, stents, instruments, etc.) inside the patient’s body. Its challenges, however, include the 2D projective nature of the images and poor soft tissue contrast. The integration of other imaging modalities during real-time guidance by means of multimodal three-dimensional (3D) image fusion (IF) can address these challenges, combining the strengths of different modalities. With the fusion packages available on commercial XR systems, allowing merging of live XR fluoroscopy with pre-interventionally derived patient-specific 3D models [1–6], real-time 3D transesophageal echocardiography (TEE) [7, 8], or virtual anatomy derived from electroanatomic mapping [9, 10], promising results have been shown for the guidance of transvascular catheter interventions, as well as during mapping and ablation of complex arrhythmias. Although IF has been proven advantageous for providing 3D anatomy, reduction of radiation exposure, increasing procedural safety and efficacy, and improved outcome [3, 6, 10–12], a wide-spread application is hindered by: (1) fusion packages limitation to a single or restricted number of applications and noncompatibility in data transfer between different vendors or even software packages of the same vendor; (2) only basic functionality including 3D volume segmentation, manual registration, and real-time image fusion; (3) static nature of the anatomic models and non-deformable rigid registration, potentially causing overlay inaccuracy; (4) usage of proprietary localization systems, which may significantly increase the costs of the intervention, require additional equipment in the intervention space, and are partly only applicable with dedicated proprietary catheters, limiting the flexibility of catheter choice during the intervention.

Since years, multiple independent research groups try to improve current IF visualization [13] and introduce more advanced guidance techniques, such as e.g., automatic cross-modal image registration [14–18], image-based tracking [14, 19], automatic compensation of heartbeat and respiratory motion [20–22] to overcome these specific issues. To introduce these techniques into a product functionality is, however, not easy due to a proprietary character of commercial XR systems. Obtaining real-time image and position-tracking data from commercial imaging systems for research purposes appears a challenging task.

Indicating the great interest of this topic, several open access software libraries and toolkits (PLUS [23], SynchroGrab [24], MITK-US [25], IGSTK [26]) providing the basic components necessary to develop its own image-guided system with defined interfaces for a number of tracking and imaging devices, particularly in the field of ultrasound (US)-guided interventions, and research platforms dedicated to intraoperative navigation with US imaging (CustusX [27]) have been emerged to the open science community for research purposes in several different clinical procedures. Whereas big progress has been made in the field of US-guided interventions and electromagnetic tracking of the instruments inside the body [21, 28–30], there is a wide spectrum of clinical procedures performed under XR fluoroscopy-guidance exclusively without intraoperative US imaging. Despite the fact that XR fluoroscopy accounts for more than 90% of intraoperative imaging [26], it is often challenging for the interventional radiologist to mentally register 2D projection images provided by XR to the 3D patient anatomy, introducing ambiguity and inaccuracies in the procedures. Thus, any additional 3D information which can be used to guide these procedures would be helpful and is highly appreciated.

In this paper, we introduce 3D-XGuide as an open-source software system dedicated to merging and visualization of the information from pre-interventional tomographic imaging and XR fluoroscopy in a common coordinate system, providing full flexibility on arbitrary C-arm angulation, zooming, and floating table manipulation. The offered core functionality (multimodal data input, projective geometry calculation, visualization, and user interaction) and advanced functionalities (3D reconstruction of working points, basic algorithms on landmark registration, motion compensation, and catheter tracking) offer the basis for further research in the field of XR-guided interventions. As it was previously demonstrated by our group [31], exemplarily, an interface to a clinical biplane XR system (Allura Xper, Philips Healthcare, Best, The Netherlands) is realized utilizing video capture devices connected to the live output ports of the XR system allowing seamless retrieval of both, the XR image data and system geometry settings from live video signal.

We provide a link to the source code https://github.com/ExCaVI-Ulm/3D-XGuide available under the BSD license. The actual software release on GitHub includes the standalone application built for 64-bit Windows 10 system with all necessary software dependencies and test phantom dataset provided for quick start. User community contributions for further development of the software are highly appreciated.

## System description

### Required hardware

3D-XGuide can be used as a pure software solution, handling the input data according to DICOM standards, fully independent on the XR system manufacturer. For the live functionality video for windows compatible capturing devices, e.g., Epiphan DVI2USB 3.0™,^1^ can be connected to the live output ports of the clinical fluoroscopy system. The current implementation of the video capture card application interface allows interfacing with clinical biplane FD20/10 and monoplane FD10 fluoroscopy systems (Allura Xper, Philips Healthcare, Best, The Netherlands), providing live video signals of 1280 × 1024 pixels resolution displaying XR image and system geometry parameters.

### Software architecture and interfaces

3D-XGuide is implemented in C++, using open-source software libraries described below. The Visualization Toolkit (VTK)^2^ is used as a core of the suggested software system. It provides an extensive framework supporting a wide variety of visualization algorithms for fully interactive 3D scene rendering and data processing. Further, easy configuration and connection to video signal sources are available as part of VTK’s application programming interface (API). Graphic User Interface (GUI) has been implemented with the Qt^3^ application framework and widget toolkit. Grassroots DICOM (GDCM)^4^ and DICOM for VTK^5^ are used for interfacing and managing DICOM files and DICOM metadata. Open Source Computer Vision Library (OpenCV)^6^ is used for image processing and tracking purposes. CMake is used for software configuration and build process orchestration.

Table 1 gives an overview of used open-source libraries with the respective version and license information included.

**Table 1:** Current system configurations

OS	CMake	Compiler	VTK	Qt	GDCM	VTK-DICOM	OpenCV
Win10	3.16.2 (BSD 3-Clause)	Visual Studio 2015	8.2.0 source (BSD 3-Clause)	5.6.0 source (GNU LGPL v.3.0)	2.6.0 source (Apache v.2.0)	0.8.9 source (BSD 3-Clause)	3.0 source (BSD 3-Clause)
Win7	3.3.0 (BSD 3-Clause)	Visual Studio 2015	6.2.0 source (BSD 3-Clause)	5.5.0 source (GNU LGPL v.3.0)	2.6.0 source (Apache v.2.0)	0.8.6 source (BSD 3-Clause)	3.0 source (BSD 3-Clause)

**Fig. 1:**
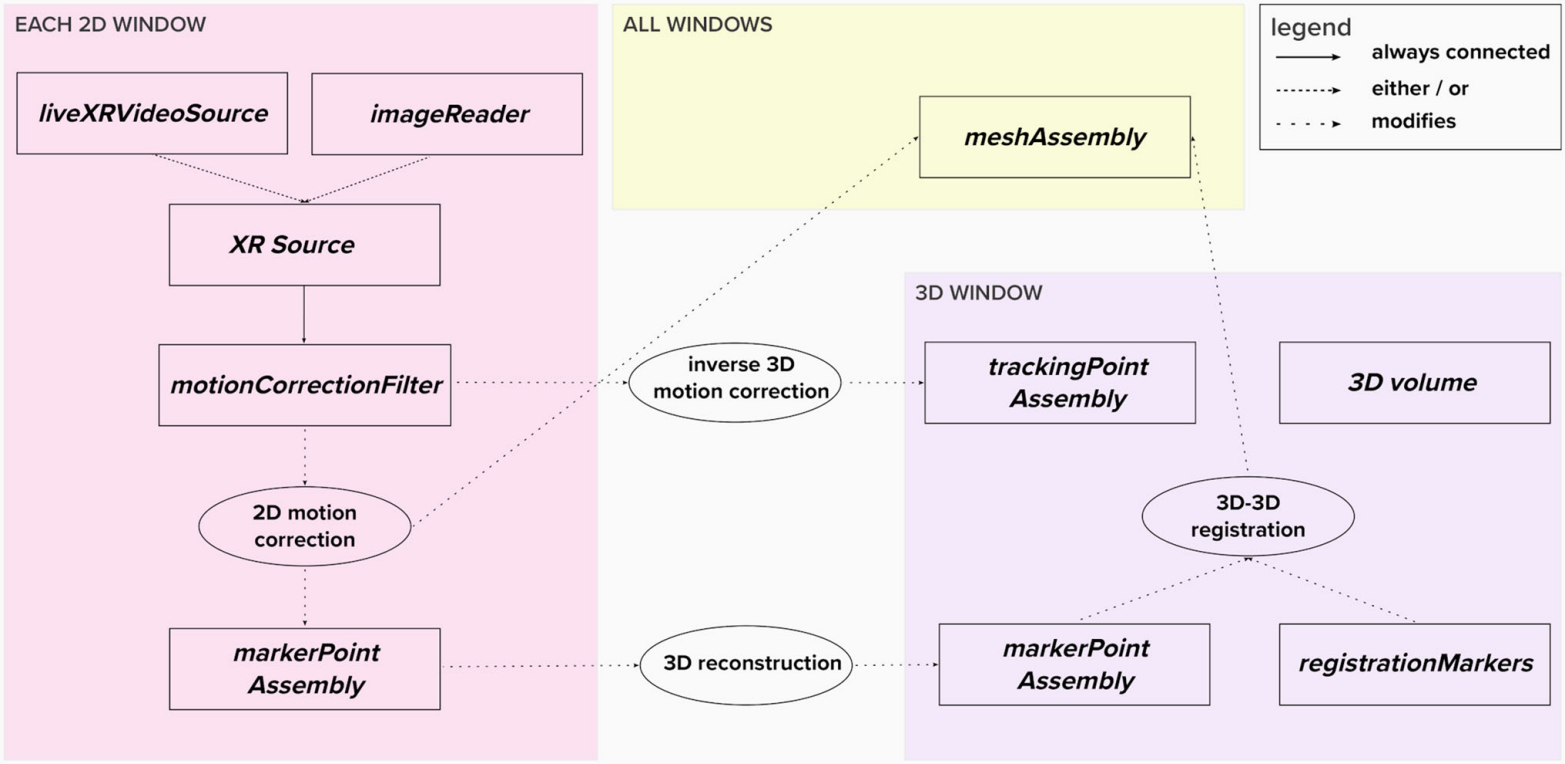
3D-XGuide data processing pipeline

3D-XGuide relies on VTK visualization pipeline architecture. The data processing pipeline is shown in Fig. 1 with implemented components being discussed below.

Following major components are implemented in the 3D-XGuide:

- *Calculation of the projection geometry and transformations* between coordinate spaces as a foundation for 2D-3D image fusion and 3D reconstruction of the target points "X-Ray projection imaging".
- *Paired-point rigid body registration* by manually identification of corresponding point sets in two spaces/modalities for transformation calculation by least squares fit implemented in VTK.
- *Motion compensation and catheter tracking algorithms* implemented as separate processing filters to be connected within the pipeline. OpenCV methods for normalized cross-correlation are used for interactive template matching-based 2D and 3D filtering. Motion compensation in 2D space is achieved by tracking the structure, which reflects the motion pattern in XR images, transferring the extracted motion vector on the initial registration, and adapting the 2D model overlay position accordingly. Catheter tip tracking in two 2D projection images and its reconstruction in 3D, combined with compensation of the 3D position with the extracted 3D motion vector allows motion-compensated catheter tracking within the static 3D model.

Moreover, the video capture card application interface is implemented for live operation to obtain the XR image data and imaging geometry of the XR system. The VTK API is used for video signal capturing and combined with a self-implemented character recognition method for the extraction of the geometry parameters from the live video signal "Obtaining live XR geometry".

### Data representation

To ensure seamless pipeline execution, the data representations and types of information within the pipeline need to be specified. MRI and/or CT images are handled in DICOM format as a series of 2D slices or 3D volume image. For converting DICOM directory of MRI/CT slices into 3D volume image ITK-SNAP^7^ tool version 2.4 [32] has been used. The 3D surface models (meshes) are represented in a generalized polygonal VTK data file format (both binary or ASCII are accepted).

Depending on the operation mode, in which the software system is used, the XR data are handled in different formats. (1) In retrospective mode, an XR run in original DICOM format (belonging to one of the XR Media Storage Standard SOP classes) can be read as a series of frames and resampled on per-frame basis for visualization. The GDCM reader is then used to extract all XR system geometry parameters needed for calculation of the respective transformations from DICOM tags in the metadata specified for the whole run. (2) For the live operation, the video signals from live DVI output ports of the XR system are captured in 24-bit RGB format with VTK video for windows video digitizer. 2D RGB images are then converted to grayscale for subsequent image processing and character recognition "Obtaining live XR geometry". (3) On demand, each captured frame can be written to hard disk as derived DICOM image and can be used for registration/refinement of the registration during live operation or in retrospective mode. Writing of DICOM files is realized using vtkDICOMWriter. Since currently the generator for XR dataset is not supported there, as workaround metadata attributes belonging to CT imaging modality are being populated with the recognized XR geometry parameters for each captured frame.

## Methods

### X-ray projection imaging

Projection of the 3D patient anatomy obtained out of pre-interventional tomographic imaging onto the 2D fluoroscopic images involves determining the transformation of the coordinate space of the 3D data into the coordinate space of 2D fluoroscopy data. In contrary to the old image-intensifier-based XR fluoroscopy systems, which produce distortions in the projection images, depending on the orientation and position of the XR gantry [33, 34], the use of modern flat-panel XR detectors enables image registration for any view direction without any complex geometry or distortion correction.

**Fig. 2:**
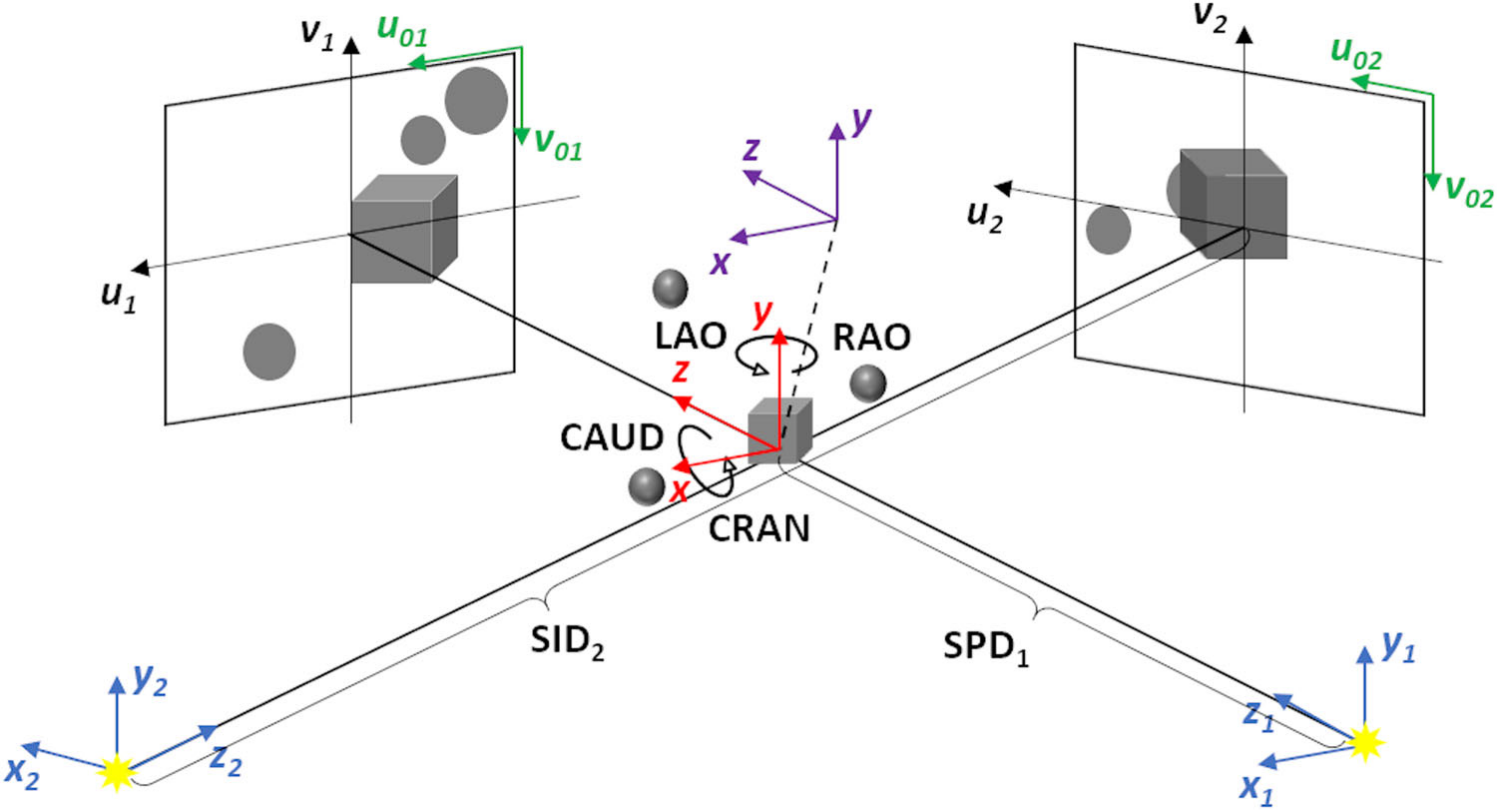
Projection geometry of a biplane X-ray system with corresponding coordinate systems (CS) indicated: world coordinate system (WCS) with the origin aligned with the XR system’s iso-center (red), patient table CS (purple), source coordinate systems (SCS) fixed in the point XR radiation source (blue), detector coordinate system (DCS) defined as the center of the detector (black), image coordinate system (ICS) with the origin in the upper left corner of the image (green). The orientation of the imaging C-arm relative to the WCS is defined by the primary (LAO(“ + ”)/RAO(“−“)) and secondary (CRAN(“ + ”)/CAUD(“−“)) angles. SID and SPD denotes source-image distance and source-to-patient distance for either C-arm

In general, the projection of a 3D object onto the 2D image plane can be described by a simple pinhole camera model, in which the object is positioned between the focal point and the image plane (Fig. 2) and is represented by a 3 × 4 homogenous projection matrix **P**, as described elsewhere [35–37]:

1 $$ {\varvec{P}} = \user2{ }\left[ {\begin{array}{*{20}c} {\frac{{\sqrt 2 \cdot {{n}}_{{{u}}} \cdot {{SID}}}}{{{{FD}}}}} & 0 & { - \frac{{{{n}}_{{{u}}} }}{2}} \\ 0 & { - \frac{{\sqrt 2 \cdot {{n}}_{{{v}}} \cdot {{SID}}}}{{{{FD}}}}} & {\frac{{{{n}}_{{{v}}} }}{2}} \\ 0 & 0 & 1 \\ \end{array} } \right] \cdot \left[ {\left( {\begin{array}{*{20}c} \ddots & {} & {} \\ {} & {{\varvec{R}}_{{{\varvec{PA}}}} {\varvec{R}}_{{{\varvec{SA}}}} } & {} \\ {} & {} & \ddots \\ \end{array} {\bigg|}\begin{array}{*{20}c} { - {{t}}_{{{x}}} } \\ { - {{t}}_{{{y}}} } \\ { - {{SPD}} - {{t}}_{{{z}}} } \\ \end{array} } \right)} \right] $$

Matrix **P** is the product of a 3 × 3 matrix representing the perspective projection and a 3 × 4 matrix describing the orientation of the imaging system relative to the world coordinate system. ${{n}}_{{{u}}}$ and ${{n}}_{{{v}}}$ are the image dimensions in pixels, *SID* denotes the source-image distance, and *FD* is the diagonal measurement of the detector. The 3 × 4 matrix results from the multiplication of the matrices describing the primary (${\varvec{R}}_{{{\varvec{PA}}}}$) and secondary (${\varvec{R}}_{{{\varvec{SA}}}}$) angulations defined as depicted in Fig. 2, and translation vector, which is given by the coordinates of the moving interventional table $\left[ {{{t}}_{{{x}}} ,{ t}_{{{y}}} ,{{t}}_{{{z}}} } \right]$ and source-to-patient distance (SPD) being fixed for each specific C-arm. All indicated distances are measured in millimeters.

For on-the-fly update of the 3D model to x-ray registration for any orientation of the XR gantry and translation of the interventional table, the projection matrix is continuously updated with the actual imaging geometry parameters (primary and secondary angles, longitudinal/lateral/vertical table positions, SID, FD).

Moreover, given initial XR fluoroscopy system calibration, with the accurate knowledge of the projection geometry, including the location and orientation of the focal spot and detector, three-dimensional reconstruction of a certain point can be performed from projection images obtained at different view orientations using epipolar constraints, as described elsewhere [38].

### Obtaining live XR geometry

**Fig. 3:**
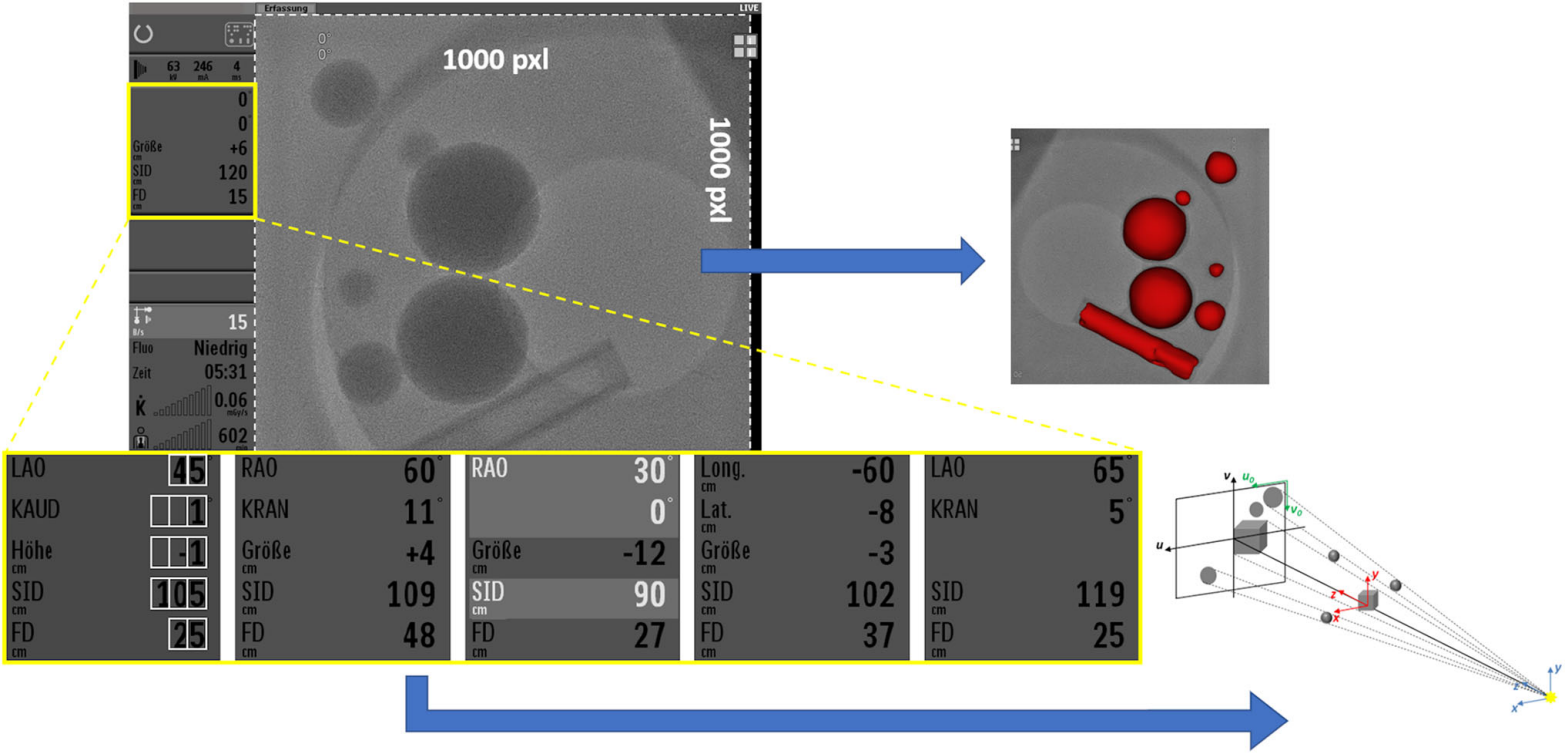
Captured live XR video signal of 1280 × 1024 pixels resolution. The fluoroscopic image has fixed size of 1000 × 1000 pixels and position within the template and is used for the visualization. The panel to the left includes restricted number of geometry settings displayed at specific pixel locations (yellow box). Five close-ups on the bottom show different possible combinations of the displayed geometry values. These values are used for calculation of transformations between the coordinate systems. On the first close-up, thirteen pixel matrices analyzed during character recognition are indicated as white boxes

Whereas all XR system geometry parameters needed for calculation of the transformations described above can be retrospectively read from original DICOM metadata, for live functionality this information has to be extracted in a different way. Fortunately, 1280 × 1024 pixels live video signal of the XR system provides all related geometry parameters, displayed at specific pixel locations within a single configuration panel located on the left-hand side of the XR image of fixed matrix size of 1000 × 1000 pixels. Figure 3 represents different possible combinations of the geometry settings (including primary/secondary angulations displayed in degrees, longitudinal/lateral/vertical table positions, source-image distance (SID) and FD displayed in centimeters) corresponding to the frontal and lateral C-arms.

To obtain the complete geometry set for either C-arm thirteen positions within the well-defined templates need to be analyzed (Fig. 3, first close-up, white boxes). The Frobenius norm of the pixel matrix representing each single possible character (digits from 0 to 9, “−/+ ” signs, and “empty”) is calculated. Prior to calculation of the norm, the pixel matrix representing each specific character is converted to binary values to avoid any luminance and contrast dependency, thus allowing to uniquely identify all required values. Additional analysis of few distinct pixels allows to distinguish between: table coordinates and angulation (they are displayed at the same positions within the template and differ by the presence/absence of the degree sign), “LAO” and “RAO,” as well as “CRAN” and “CAUD” orientations (please consider German literation) to derive the sign of the C-arm angulation.

Unfortunately, table position, SID, and FD are provided by the vendor in full centimeters and the angulation in full degrees, introducing a maximal rounding error of ± 5 mm and ± 0.5° for a single geometry parameter propagating for the 2D projections on the detector plane. This issue limits the accuracy of the calculated transformations and need to be closer investigated.

### Live operation evaluation

The following two aspects relevant for the live operation of the system were evaluated:

1. performance of pipeline steps execution;
2. accuracy of registration and image fusion.

The evaluation of pipeline steps execution performance for live operation was done based on an average of at least a hundred executions separate for monoplane and biplane operation.

The accuracy of registration and image fusion was assessed by means of a phantom experiment. A custom-designed MRI/XR phantom consisting of glass spheres of three different sizes (6, 10, and 20 mm in diameter) and a single glass tube embedded in agarose gel was imaged with MRI at an isotropic resolution of 0.5 mm^3^. Segmentation of 3D MRI volume was performed using EP Navigator R5.1.1.4 tool (Philips Healthcare, Best, The Netherlands) and converted to VTK polygonal data file using MATLAB. Subsequently, the phantom was imaged on the biplane XR system at different geometry settings. The XR data were recorded with 3D-XGuide for accuracy estimation.

**Fig. 4:**
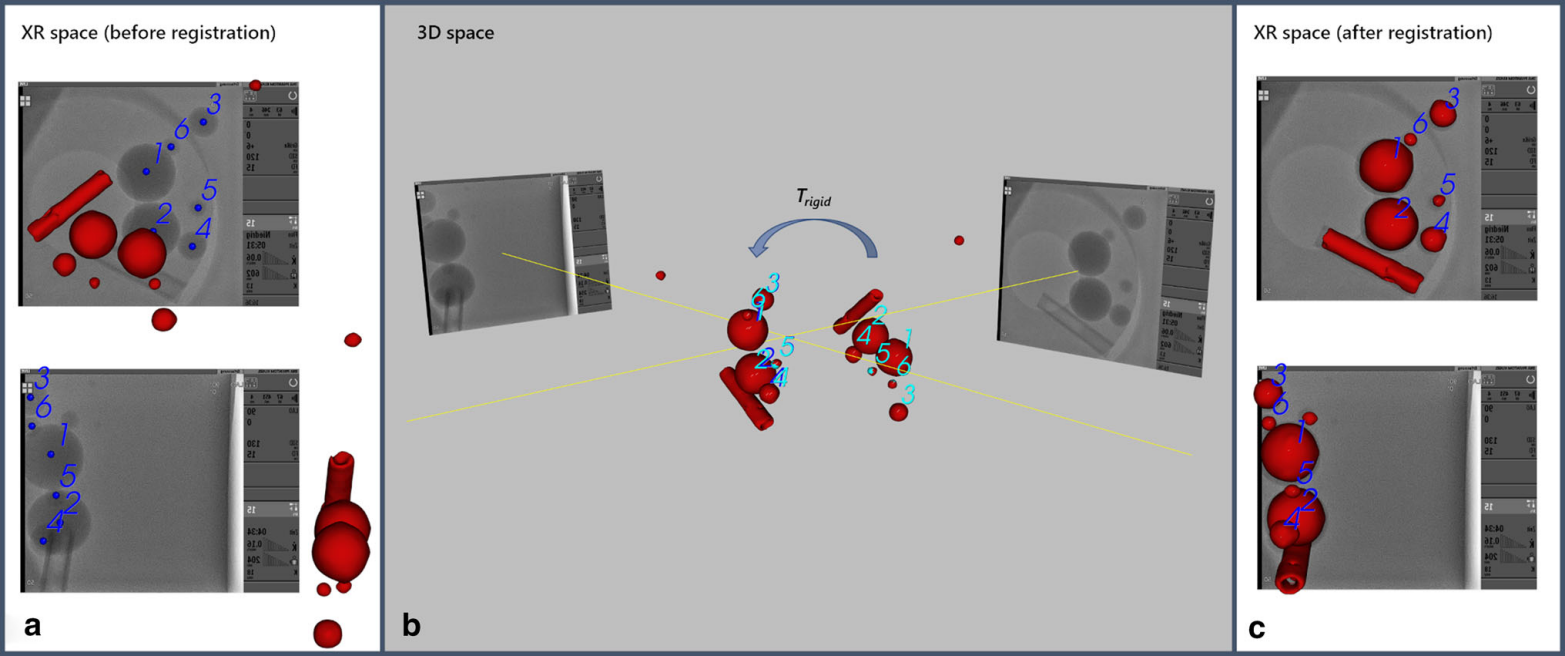
Registration between 3D MRI and 2D XR space: **a** two sets of six corresponding 2D points are manually identified in the center of respective glass spheres (blue) in two orthogonal XR views; **b** 3D scene with 3D points reconstructed from corresponding 2D points identified in each XR view (blue) and corresponding 3D MRI points (turquoise) before T_rigid_ registration (sets of blue and turquoise points are spread in space) and after (sets of blue and turquoise points are aligned); **c** result of the registration in XR space as 2D overlays in two orthogonal views

Four to six marker positions in the center of respective glass spheres [2D pairs in two respective XR projection images (Fig. 4a), 3D in MRI (Fig. 4b)] were manually identified in both acquired datasets. The 3D reconstruction method implemented in 3D-XGuide was then applied to reconstruct the 3D position of each paired XR point (*P*_XR_). Implemented paired-point 3D-3D rigid body registration was then performed between the MRI marker positions (*P*_MRI_) and 3D positions of points reconstructed from its two-dimensional XR projections *P*_XR_ for each individual case of investigated geometry setting. The transformation matrix *T*_rigid_ between two spaces was retrieved and the registration accuracy for a single geometry setting was assessed in terms of root-mean-squared error (RMSE) between the transformed *P*_MRI_ marker positions and reconstructed 3D *P*_XR_ positions according to:

2 $$ {\text{RMSE}} = \sqrt {\frac{1}{n} \mathop \sum \limits_{i = 1}^{n} \left( {T_{{{\text{rigid}}}} P_{{{\text{MRI}}}}^{i} - P_{{{\text{XR}}}}^{i} } \right)^{2} } $$

The resulting registration accuracy was averaged among all investigated combinations of markers and geometry settings.

The accuracy evaluation of the image fusion following initial registration was performed visually inspecting the difference between the 3D model and its projection.

The impact of the error introduced by the rounding of the displayed geometry parameters on the accuracy of the calculated transformations was investigated for an angulation rounding error $\epsilon_{a}$ of 1° and table position rounding error $\epsilon_{t}$ of 1 cm as maximal possible deviation between two images with the same displayed configuration values. The values were calculated as maximal possible deviation from the optical axis (pointing from source towards image center) from the geometric relations obtained at the largest projection magnification (achieved at maximal *SID*):

3 $$ \epsilon_{a} = \sqrt {b^{2} + \left( {{\text{SID}}_{\max } - {\text{SPD}}} \right)^{2} - 2b \cdot \left( {{\text{SID}}_{\max } - {\text{SPD}}} \right) \cdot \cos \left( {1^{^\circ } } \right)} $$

with $$b = \sin \left( {90^\circ } \right) \cdot \frac{{{\text{SID}}_{\max } - {\text{SPD}}}}{{\sin \left( {90^\circ - 1^\circ } \right)}}$$.

4 $$ \epsilon_{t} = \frac{{10\,{\text{mm}}}}{{{\text{SPD}}}} \cdot {\text{SID}}_{\max } $$

The reliability of the introduced character recognition method against possible input dependent pixel hue values variations was additionally investigated in a test setup simulating intensity variations during video capturing.

## Results

### Geometry extraction from live XR fluoroscopy

XR system geometry settings could be extracted correctly in 100% of cases, proving the robustness of the implemented character recognition approach.

### System performance

Table 2 summarizes the averaged execution times for the pipeline steps, involved in the live operation. Compared to the VTK-native methods for video frame capturing, updating, and rendering, the geometry extraction can be neglected for the entire system performance. In general, a seamless operation at 30 frames-per-second (fps) could be achieved.

**Table 2:** Averaged execution times with standard deviations of the pipeline steps in the live operation

Capture (ms)		Pipeline information update (ms)	Geometry extraction (ms)	Rendering (ms)	Total time (ms)	Total rate (fps)
16.1 ± 6.1	8.7 ± 3.6		0.2 ± 0.4	8.8 ± 5.3	33.7 ± 8.9	~ 30

For biplane operation, all performance measurement results have to be doubled since frame capturing on two devices is currently not parallelized and the pipeline is executed successively, yielding a maximum of 15 fps.

### Accuracy of registration and image fusion

Figure 4 demonstrates the resulting marker-based 3D-3D registration in 2D XR and 3D MRI space. The fusion of 3D volume overlay with two XR projection images acquired at LAO 0° and LAO 90° orientations is shown before and after registration on Fig. 4a, c, respectively. On Fig. 4b, the MRI volume before and after 3D rigid-body transformation is shown.

All investigated combinations of geometry settings for registration are summarized in Table 3.

**Table 3:** Overview of the geometry settings taken into account for calculation of the respective transformations during co-registration in each individually investigated case. Root-mean-squared errors (RMSE) for each set are provided in the last column

Set	Number of paired registration points	C-arm	Primary angulation	Secondary angulation	Table (cm): long, lat, vert	SID (cm)	FD (cm)	RMSE (mm)
Set 1	6	Frontal	LAO 0°	CAUD 0°	− 64, − 7, 6	120	15	0.31
		Lateral	LAO 90°	CAUD 0°	− 64, − 7, 6	130	15	
Set 2	5	Frontal	RAO 26°	CAUD 0°	− 64, − 7, 6	120	15	0.36
		Lateral	LAO 45°	CAUD 0°	− 64, − 7, 6	130	15	
Set 3	6	Frontal	RAO 45°	CAUD 0°	− 64, − 2, 6	120	15	0.32
		Lateral	LAO 45°	CAUD 0°	− 64, − 7, 6	130	15	
Set 4	5	Frontal	RAO 45°	CAUD 0°	− 63, − 2, 6	120	15	0.31
		Lateral	LAO 45°	CAUD 0°	− 64, − 7, 6	130	15	
Set 5	4	Frontal	LAO 0°	CAUD 25°	− 63, − 7, 6	120	15	0.39
		Lateral	LAO 85°	CRAN 29°	− 63, − 7, 6	130	15	

The mean error over all investigated combinations of markers and geometry settings resulted in 0.34 mm, indicating an accuracy in the order of spatial resolution of the acquired 3D volume.

**Fig. 5:**
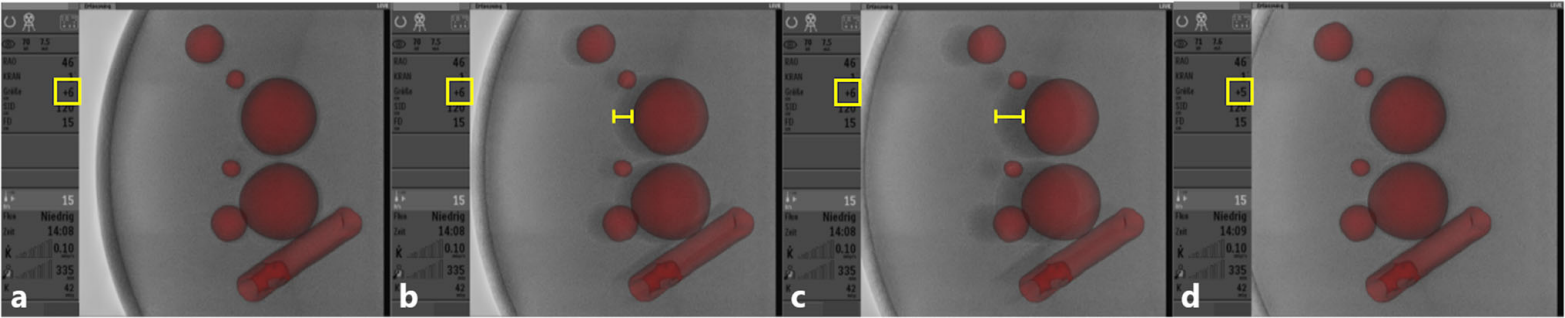
Misalignment of the model overlay due to the rounding of the displayed table coordinates. Images are recorded consequently during movement of the table in vertical direction only from the displayed value of + 6 (**a**–**c**) to the displayed value of + 5 (**d**). All other geometry settings are kept constant

After initial co-registration, the 3D model automatically followed the system geometry correctly for the whole range of possible system settings. Despite the fact that all system geometry settings could be extracted correctly and time synchronously, the rounding errors in the displayed table coordinates and angles limited the accuracy of the calculated transformations. Following initial registration, this leads to a misalignment of the superimposed model during image fusion in case the displayed values do not perfectly match the real settings. A mismatch between the model overlay and its projection during continuous movement of the interventional table in vertical direction from displayed value “+ 6” (Fig. 5a–c) to displayed value “+ 5” (Fig. 5d) can be exemplarily appreciated in Fig. 5.

The maximal error along the centerline was estimated to be 14.8 mm for the frontal C-arm and 16.99 mm for the lateral C-arm for a maximal rounding error of 1 cm in the displayed table coordinate. For the angulation, maximal displacements result in 6.8 mm (frontal) and 9.4 mm (lateral). The error will increase with the increasing distance to the centerline and iso-center and can accumulate.

Errors introduced by the rounding of the FD values can be avoided by using specific pixel spacing values obtained from original DICOM metadata for each individual FD setting. These pixel spacing values were obtained for eight FDs available on the frontal C-arm ranging from 15 to 48 cm, and three FDs ranging from 15 to 25 cm for the lateral and monoplane C-arm. Due to the system geometry, the rounding error in displayed SID does only contribute to deviation in magnification and no obvious displacement or misregistration was observed.

## Discussion

This paper introduces 3D-XGuide as a software system for multimodal fusion and navigation guidance in the catheterization laboratory. Compared to commercial tools aiming for propriety solutions, the introduced system is open-source available under the BSD 2-Clause license.

The contribution of this work is twofold. First, the introduced system provides a common software basis for image fusion of the pre-intentionally acquired 3D anatomy and XR fluoroscopy. 3D-XGuide provides an open-source implementation of the basic functionalities required for image-guided interventions on X-ray systems. With the underlying VTK pipeline implementation, 3D-XGuide allows to combine, extend, modify, and optimize the modules implemented in the pipeline for specific research interests, and thus provides a basis for the rapid prototyping of new approaches and algorithms in the field of XR fluoroscopy-based guidance.

Second, the video capture card application interface supports the evaluation of the developed algorithms in the real clinical settings by allowing import of the XR image data and system geometry settings from live video output of the XR system. The idea of obtaining live XR images by video capturing is not new. However, to obtain the system geometry, position-tracking systems need to be integrated, requiring additional hardware in the interventional space. Alternatives like image-based tracking methods (fiducial marker-based or markerless tracking [39–41]) have been suggested, but the required 2D-3D registration limits its accuracy. Deriving the geometry from the live images allows indirect usage of the vendor’s position encoders. Although adjustment of the template might be needed for XR systems of other manufacturers, the proposed character recognition method is easy to implement and is robust and time-efficient.

Although Epiphan devices perfectly match the requirements for high-resolution high-speed XR imaging, the transfer rates exceeding 30 fps monoplane operation could not be realized in the current implementation, primarily due to the rather inefficient demand-driven execution control in VTK. Moreover, due to successive pipeline execution, frames captured on the lateral C-arm output are delayed roughly by 25 ms as compared to the frontal output in case of biplane operation. This may cause additional synchronization challenges for 3D motion compensation and catheter tracking and need to be optimized in future.

Also, the rounding of the displayed geometry values needs to be taken into account. In the real clinical setting, the mismatch introduced by the rounding of the displayed table coordinates and angulations might appear less pronounced as in the phantom experiment, since due to increased scatter intraoperative XR imaging is normally not performed at maximum SID. Even though the slight mismatch may be acceptable, it limits the accuracy of the introduced approach and cannot be mitigated.

To conclude, although the current implementation is still facing some performance limitations, it is already accurately applicable for image fusion during biplane operation at constant geometry as required for procedures like e.g., pulmonary vein isolation. Moreover, full functionality on arbitrary C-arm and floating table manipulation is implemented, thus allowing the extension to other applications as long as the lack of accuracy can be tolerated.

## Conclusions

Technologies like image fusion will push the limits in image-guided interventions, not only aiding the interventionalists in better understanding and navigating the anatomy, but leading to increased procedure safety and efficacy. However, the current lack in respective open-source and open-architecture multimodal intervention guidance frameworks prevents 3D augmentation from fulfilling its potential, particularly in X-ray navigation. In this work, a 3D-XGuide software system providing a foundation for further research and development in the field of image-guided interventions is introduced. Providing the source code, we would like to encourage the scientific community to further develop and evaluate the proposed software solution towards meeting the requirements of safety–critical medical applications, to make it applicable to a variety of percutaneous interventions.

## Data Availability

The link to actual software release including executable and source code is available online under the BSD 2-Clause license: https://github.com/ExCaVI-Ulm/3D-XGuide.

## Footnotes

1 https://www.epiphan.com/products/dvi2usb-3-0/.

2 https://www.vtk.org/.

3 https://www.qt.io/.

4 https://github.com/malaterre/GDCM.

5 https://github.com/dgobbi/vtk-dicom/.

6 https://opencv.org/.

7 http://www.itksnap.org/pmwiki/pmwiki.php.
